# Cooperative Localization and Time Synchronization Based on M-VMP Method

**DOI:** 10.3390/s20216315

**Published:** 2020-11-05

**Authors:** Zhongliang Deng, Shihao Tang, Buyun Jia, Hanhua Wang, Xiwen Deng, Xinyu Zheng

**Affiliations:** School of Electronic Engineering, Beijing University of Posts and Telecommunications, Beijing 100876, China; dengzhl@bupt.edu.cn (Z.D.); jiabuyun@bupt.edu.cn (B.J.); whh0710@bupt.edu.cn (H.W.); dengxiwen@bupt.edu.cn (X.D.); buptzxy@bupt.edu.cn (X.Z.)

**Keywords:** multi-variational message passing (M-VMP), factor graph (FG), second-order Taylor expansion, cooperative localization, joint estimation of position and clock

## Abstract

Localization estimation and clock synchronization are important research directions in the application of wireless sensor networks. Aiming at the problems of low positioning accuracy and slow convergence speed in localization estimation methods based on message passing, this paper proposes a low-complexity distributed cooperative joint estimation method suitable for dynamic networks called multi-Gaussian variational message passing (M-VMP). The proposed method constrains the message to be a multi-Gaussian function superposition form to reduce the information loss in the variational message passing algorithm (VMP). Only the mean, covariance and weight of each message need to be transmitted in the network, which reduces the computational complexity while ensuring the information completeness. The simulation results show that the proposed method is superior to the VMP algorithm in terms of position accuracy and convergence speed and is close to the sum-product algorithm over a wireless network (SPAWN) based on non-parametric belief propagation, but the computational complexity and communication load are significantly reduced.

## 1. Introduction

In recent years, wireless sensor network has been widely used in agriculture, warehousing, production safety, emergency rescue and other fields [1]. The information which sensors collected and transmitted is valuable when combined with the sensors’ location [2,3]. Therefore, location awareness of wireless sensor networks has become one of the most important directions in the development of wireless sensor networks. Global Navigation Satellite System (GNSS) is able to provide location information for sensor nodes, but it is difficult to apply on sensor networks due to high power consumption and high cost [2,3,4]. Furthermore, the poor signal penetration capabilities of GNSS lead to inadequate location information in indoor scenes. Cooperative localization can overcome these problems through ranging and exchanging locations between neighbor nodes [5]. Over the past decade, cooperative localization in wireless sensor networks has drawn considerable attention.

In the recent ten years, cooperative positioning in wireless sensor networks has been widely concerned. The multidimensional scaling (MDS) algorithm [6,7] calculates the shortest path distance between nodes according to the connectivity of the network or distance measurement. Then, the relative coordinate diagram of all nodes is constructed by using the multidimension scale algorithm. Finally, the relative coordinate diagram is transformed into an absolute coordinate graph according to the coordinate of the anchor node. The semi-definite programming (SDP) algorithm [8,9] represents geometric constraints among nodes as a set of linear matrix inequalities, then combines the inequality into a semi-definite programming problem and obtains the location of each node by global optimization. The distance-vector (DV) hop algorithm [10,11] firstly measures the minimum number of hops between the nodes to be located and each anchor node. Then, the average distance of each hop is determined according to the number and coordinate between anchor nodes. After the jump number is converted into distance, the position of the node to be located is obtained according to the trilateral measurement method. The Approximate Perfect Point-In-Triangulation (APIT) algorithm [12,13] firstly selects three anchor nodes connected with the node to be located, and then judges whether they are in the triangle composed of anchor nodes according to the pit test, and then repeats the pit test with a different anchor node combination. Finally, the center of mass position of these triangles is taken as the coordinates of the nodes to be located. The SPAWN algorithm [14] decomposes the posterior probability density function of position variables and represents it as a factor graph. Then, the message is transmitted by particles on the factor graph to calculate the probability of edge distribution of each variable. Finally, position estimation is carried out according to minimum mean square error (MMSE) or map criterion. Although the above cooperative positioning algorithm has high positioning accuracy, it has high computational complexity and high communication cost, which seriously restricts the practical application.

For the above problems, a collaborative location algorithm based on factor graph and VMP is proposed in Reference [15], which has simple message form and small computation. However, Kullback-Leibler (KL) divergence is used to Gauss the non-Gaussian confidence of the nonlinear range measurement model. The first kind of convergence hypergeometric function minimization problem is introduced, which makes the calculation complexity very high. In Reference [16], a hard decision-based cooperative algorithm is proposed to alleviate the effect of the outliers. The geometric relationship between agent position and distance is used to avoid a large deviation coursed by geometric constraints. The authors of Reference [17] propose the distributed particle filtering evolved variational message passing (DPF-E-VMP) algorithm, which improves the convergence speed of positioning estimation by using distributed particle filtering (DPF), but this performance improvement is often accompanied by greater computational consumption. By combining the average consensus method and VMP algorithm, a joint self-localization tracking algorithm called cooperative localization with outlier constraints (CLOC) is proposed in Reference [18], which has better location performance than the separate self-localization algorithm. In Reference [19], KL divergence is minimized by the Newton conjugate gradient method, but the computational complexity is still high. But, the information loss caused by VMP parameterization will have a certain influence on the positioning accuracy. At the same time, the algorithm has been reduced in accuracy and convergence speed due to the clock synchronization between the nodes to be located.

In this paper, a VMP distributed cooperative localization algorithm based on multi-Gaussian is proposed. Based on the non-line of sight (NLOS) environment ranging model, the VMP message passing strategy based on multi-Gaussian is innovated, and the second-order Taylor expansion form of position and time synchronization joint estimation is derived. Its computational complexity is far less than the approximate solution based on KL divergence. The time complexity and communication cost are connected with the traditional VMP algorithm. However, the positioning accuracy and iteration speed have been greatly improved.

The remainder of this paper will be organized as follows: A two-dimensional (2D) wireless network is first established as a system model. Then, a traditional cooperative localization algorithm is introduced and leads to the method proposed in this paper. After that, simulation performance of the proposed method is investigated. Finally, concluding remarks are presented. A list of symbols that are used in the paper is given in Table 1.

## 2. System Model

The anchor nodes are always deployed at the same height, and high vertical dilution of precision means the system cannot provide reliable vertical positioning results [20], which is usually obtained by other sensors [21]. So, in this paper, a 2D dynamic wireless network is considered, which includes the anchor node with known location and synchronized local time, and the node with inaccurate location information and local time out of sync. The position vector of node i at time n is $x_{i,n}={\left[x_{i,n},y_{i,n}\right]}^{T}$, and the measured value of local clock ${\tilde{t}}_{i,n}=t_{i}\left(T_{n}\right)$. The slope of local clock at time n between node i and the external standard clock is $a_{i,n}=\left({\tilde{t}}_{i,n}-{\tilde{t}}_{i,n-1}\right)/\left(T_{n}-T_{n-1}\right)$. The local clock of all anchor nodes is synchronized with the external reference clock, i.e., $a_{i,n}=1\forall i\in S$. At time n, node i has neighbor nodes set as $N_{i,n}$, where neighbor anchor node set is $S_{i,n},$ node set to be located is $C_{i,n}$, and all communicable node pairs $\left(i,j\right)$ constitute communicable node set $\Psi_{n}$.

Considering the local clock drift, node i measures time of arrival (TOA) from neighbor node j at time n as follows:

(1) $${\tilde{\rho }}_{ij,n}=∥x_{i,n}-x_{j,n}∥+\zeta_{ij,n}+cTa_{ij,n}+\omega_{ij,n},$$

where $∥x_{i,n}-x_{j,n}∥$ is the Euclidean distance, $d_{ij,n},$ between nodes i, j, $T=T_{n}-T_{n-1}$, $\omega_{ij,n}$ is the observed measurement noise, assuming that it obeys the Gaussian distribution, i.e., $\omega_{ij,n}∼𝒩\left(0,\sigma_{d}^{2}\right)$, and $\zeta_{ij,n}$ is NLOS error, which is expressed as follows:

(2) $$\zeta_{ij,n}=\left\{\begin{array}{c} 0,\left(i,j\right)\notin \Theta_{n}\\ \lambda e^{-\lambda b_{ij,n}},\left(i,j\right)\in \Theta_{n} \end{array}\right.,$$

where $b_{ij,n}>0$ [22], λ is a constant and $\Theta_{n}$ is the set of all node pairs $\left(i,j\right)$ with NLOS error at time n. Where, $a_{ij,n}$ is the relative slope of local clock offset between nodes i,j, defined as follows:

(3) $$a_{ij,n}≜\left\{\begin{array}{c} \frac{a_{i,n}}{a_{j,n}},t_{i,n}>t_{j,n}\\ \frac{a_{j,n}}{a_{i,n}},t_{i,n}>t_{j,n} \end{array}\right.,$$

Define $x_{n}≜{\left[x_{1,n}^{T},x_{2,n}^{T},\ldots ,x_{S+C,n}^{T}\right]}^{T}$ as the position vector of all nodes, $a_{n}≜{\left[a_{1,n},a_{2,n},\ldots ,a_{S+C,n}\right]}^{T}$ as the clock offset slope of all nodes, ${\tilde{\rho }}_{n}≜{\left[\ldots ,\rho_{ij,n},\ldots \right]}^{T}$ as the range measurement in all node pairs $\left(i,j\right)\in \Psi_{n}$, and $\zeta_{n}≜{\left[\ldots ,\zeta_{ij,n},\ldots \right]}^{T}$ as the NLOS error in all node pairs $\left(i,j\right)\in \Theta_{n}$. Moreover, all the vector sets from time 1 to time n are defined as follows: ${𝓧}_{1:n}≜\left\{x_{1},x_{2},\ldots ,x_{n}\right\}$, ${𝓐}_{1:n}≜\left\{a_{1},a_{2},\ldots ,a_{n}\right\},\;{𝓟}_{1:n}≜\left\{\rho_{1},\rho_{2},\ldots ,\rho_{n}\right\},\;\;{𝓑}_{1:n}≜\left\{\zeta_{1},\zeta_{2},\ldots ,\zeta_{n}\right\}$. The goal of the algorithm is to estimate the accurate node location vector $x_{n}$ and the clock slope $a_{n}$ by measuring ${𝓟}_{1:n}$ and the information transmitted between nodes.

## 3. M-VMP Joint Estimation Algorithm

According to whether the location information is considered as a random variable, the cooperative localization algorithms are divided into two categories: non-Bayesian estimation and Bayesian estimation [5]. In non-Bayesian estimation, the location information is estimated by deterministic methods. The typical algorithms are least square (LS) [23,24] and the maximum likelihood method (ML) [25]. Bayesian estimation is based on the probability model of location information. The typical algorithms include the maximum posterior probability (MAP) estimation [26] and the MMSE method [6]. Location information node i estimates by MMSE criteria. The expression is as follows:

(4) $${\hat{𝔃}}_{i,n}=\int {𝔃}_{i,n}p({𝔃}_{i,n}|{𝓟}_{1:n})d{𝔃}_{i,n}$$

where ${\hat{𝔃}}_{i,n}$ is the estimation result and ${𝔃}_{i,n}={\left[x_{i,n},y_{i,n},a_{i,n}\right]}^{T}$ is the vector to be estimated.

### 3.1. Probability Model

Assume $x_{i,n}$ evolve according to a memory-less Gauss–Markov process, then, there are:

(5) $$x_{i,n}=x_{i,n-1}+v_{i,n}T+\omega_{i,n},$$

where $v_{i,n}$ is the average velocity of node i from time n−1 to time n, which is measured by the sensor inside nodes. $\omega_{i,n}$ is Gaussian white noise, and its covariance matrix is $diag\left\{\sigma_{i,n}^{2},\sigma_{i,n}^{2}\right\}$. Since the motions of all nodes are independent of each other, there are:

(6) $$p\left(x_{n}|x_{n-1}\right)=\prod_{i}p(x_{i,n}|x_{i,n-1})$$

(7) $$p\left(a_{n}|a_{n-1}\right)=\prod_{i}p(a_{i,n}|a_{i,n-1}),$$

(8) $$p\left({𝓧}_{1:n}\right)=p\left(x_{0}\right)\prod_{n}p(x_{n}|x_{n-1}),$$

(9) $$p\left({𝓐}_{1:n}\right)=p\left(a_{0}\right)\prod_{i}p(a_{n}|a_{n-1}),$$

where $p\left(x_{0}\right)$ is the prior distribution of all nodes’ location information at time 0, which is obtained by GNSS, base station or in other ways [27]. According to the Bayesian rule, the edge probability function in (3) is calculated by the following formula:

(10) $$p({𝓧}_{1:n},{𝓐}_{1:n}|{𝓟}_{1:n})\propto p({𝓟}_{1:n}|{𝓧}_{1:n},{𝓐}_{1:n})p\left({𝓧}_{1:n},{𝓐}_{1:n}\right)$$

The likelihood function $p({𝓟}_{1:n}|{𝓧}_{1:n},{𝓐}_{1:n})$ in (10) is decomposed into the following formula with the independent observations at different times $\rho_{ij,n}$:

(11) $$p\left({𝓟}_{1:n}|{𝓧}_{1:n},{𝓐}_{1:n})\propto \prod_{n}\prod_{\left(i,j\right)\notin \Theta_{n}}p_{LOS}(\rho_{ij,n}\right)\prod_{\left(i,j\right)\in \Theta_{n}}p_{NLOS}(\rho_{ij,n}),$$

(12) $$p_{LOS}\left(\rho_{ij,n}\right)\propto \exp\left[-\frac{{\left(\rho_{ij,n}-∥x_{i,n}-x_{j,n}∥-cTa_{ij,n}\right)}^{2}}{2\sigma_{d}^{2}}\right],$$

(13) $$p_{NLOS}\left(\rho_{ij,n}\right)\propto \exp\left[-\frac{{\left(\rho_{ij,n}-∥x_{i,n}-x_{j,n}∥-cTa_{ij,n}-\zeta_{ij,n}\right)}^{2}}{2\sigma_{d}^{2}}\right],$$

According to the above derivation, (10) is expanded to the following formula:

(14) $$\begin{array}{cc} p({𝓧}_{1:n},{𝓐}_{1:n}|{𝓟}_{1:n}) & \\ & \propto \prod_{n}\prod_{\left(i,j\right)\notin \Theta_{n}}p_{LOS}(\rho_{ij,n})\prod_{\left(i,j\right)\in \Theta_{n}}p_{NLOS}(\rho_{ij,n})\\ & \cdot \prod_{n}p\left(x_{0,n}\right)p\left(a_{0,n}\right)\prod_{i}p(x_{i,n}|x_{i,n-1})p(a_{i,n}|a_{i,n-1}), \end{array}$$

### 3.2. Factor Graph Model

**Figure 1:**
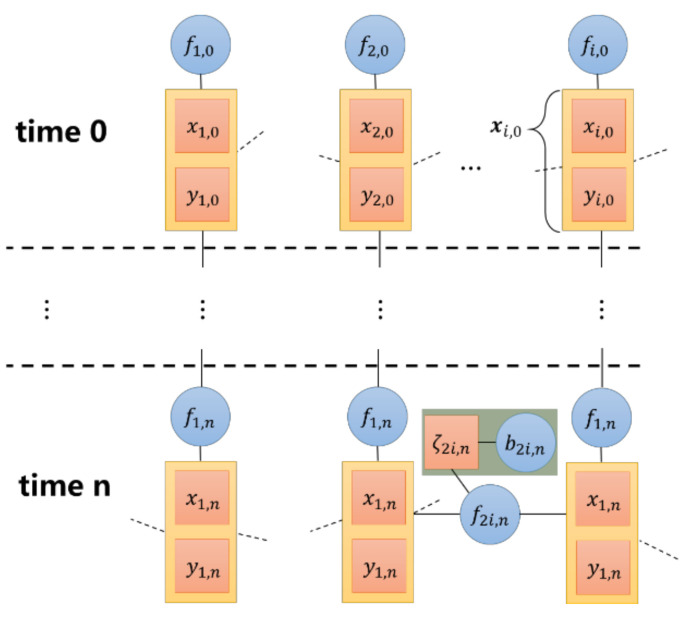
Factor graph model of variational message passing (VMP)-based cooperative localization problem. The circle represents the factor node and the square represents the variable node. $x_{i,n}={\left[x_{i,n}\;y_{i,n}\right]}^{T}$ represents the localization variable of the node to be located, $f_{i,n}$ represents the message that transmits between different times, $f_{ij,n}$ represents the distance information that the nodes transmit, $\zeta_{ij,n}$ represents the non-sight distance parameter that affects the information between nodes and $b_{ij,n}$ represents the non-sight distance error probability function that affects the non-sight distance parameter.

Factor graphs intuitively reflect the spatiotemporal relationship between variables [5]. The factor graph is a dichotomous graph, which contains two kinds of nodes: factor node and variable node. Variable node represents the information to be evaluated, and factor node represents the messages passed between variable nodes. The schematic diagram of cooperative localization is shown in Figure 1.

Factor nodes are further assumed as follows:

(15) $$f_{i,0}=p\left({𝔃}_{i,0}\right),f_{i,n}=p({𝔃}_{i,n}|{𝔃}_{i,n-1}),$$

(16) $$f_{ij,n}=\{\begin{array}{c} p_{LOS}\left(\rho_{ij,n}\right),\left(i,j\right)\notin \Theta_{n}\\ p_{NLOS}\left(\rho_{ij,n}\right),\left(i,j\right)\in \Theta_{n} \end{array},$$

(17) $$\zeta_{ij,n}=p\left(b_{ij,n}\right),$$

According to the above definition, edge probability of localization is clearly calculated in the factor graph. Next, a localization algorithm based on the VMP will be introduced.

### 3.3. VMP-Based Localization Algorithm

VMP uses the exponential model to deliver messages, which greatly reduces communication consumption. According to the VMP message rules based on the factor graph proposed in Reference [28] and the assumptions in Section 3.2, the message delivered from factor node to parameter node at time n is as follows:

(18) $$\mu_{f_{ij,n}\to {𝔃}_{i,n}}\left({𝔃}_{i,n}\right)=\exp\left\{\int \;\mu_{b_{ij,n}\to f_{ij,n}}\left(b_{ij,n}\right)\mu_{{𝔃}_{j,n}\to f_{ij,n}}\left({𝔃}_{j,n}\right)\ln f_{ij,n}\left({𝔃}_{i,n},{𝔃}_{j,n}\right)d{𝔃}_{j,n}\right\},$$

(19) $$\mu_{f_{i,n}\to {𝔃}_{i,n}}\left({𝔃}_{i,n}\right)=p\left({𝔃}_{i,n}|{𝔃}_{i,n-1}\right),$$

(20) $$\mu_{f_{ij,n}\to b_{ij,n}}\left(b_{ij,n}\right)=\exp\left\{\int \;\mu_{{𝔃}_{i,n}\to f_{ij,n}}\left({𝔃}_{i,n}\right)\mu_{{𝔃}_{j,n}\to f_{ij,n}}\left({𝔃}_{j,n}\right)\ln f_{ij,n}\left({𝔃}_{i,n},{𝔃}_{j,n}\right)d{𝔃}_{j,n}\right\},$$

where the cooperative messages $\mu_{{𝔃}_{j,n}\to f_{ij,n}}\left({𝔃}_{j,n}\right)$ are divided into two categories: one is related to the neighbor anchor node $j\in S_{i,n}$, and the other is related to the neighbor node $k\in C_{i,n}$ to be located. The calculations are performed below:

(21) $$\mu_{f_{ij,n}\to {𝔃}_{i,n}}\left({𝔃}_{i,n}\right)\propto 𝒩\left(\rho_{ij,n}|∥x_{i,n}-x_{j,n}∥,\;\sigma_{d}^{2}\right)$$

(22) $$\mu_{f_{ij,n}\to {𝔃}_{i,n}}\left({𝔃}_{i,n}\right)=\text{exp}\{E_{b(x_{k})}[𝒩(\rho_{ik,n}|∥x_{i,n}-x_{k,n}∥,\;\sigma_{d}^{2})]\},$$

where $E_{f\left(\cdot \right)}\left[g\left(\cdot \right)\right]$ means the mean of g(⋅) with respect to f(⋅). The belief of ${𝔃}_{i,n}$ is obtained as follows:

(23) $$\begin{array}{ll} b\left({𝔃}_{i,n}\right) & =\frac{1}{Z}\mu_{f_{i,n}\to {𝔃}_{i,n}}\left({𝔃}_{i,n}\right)\prod_{j\in S_{i,n}}\mu_{f_{ij,n}\to {𝔃}_{i,n}}\left({𝔃}_{i,n}\right)\prod_{k\in C_{i,n}}\mu_{f_{ik,n}\to {𝔃}_{i,n}}\left({𝔃}_{i,n}\right)\\ & \propto \frac{1}{Z}𝒩\left({𝔃}_{i,n}|{𝔃}_{i,n-1}+v_{i,n}T,\;\sigma_{i,n}^{2}\right)\prod_{j\in S_{i,n}}𝒩\left(\rho_{ij,n}|∥x_{i,n}-x_{j,n}∥,\;\sigma_{d}^{2}\right)\\ & \times \prod_{k\in C_{i,n}}\exp\left\{E_{b\left(x_{k}\right)}\left[𝒩\left(\rho_{ik,n}|∥x_{i,n}-x_{k,n}∥,\;\sigma_{d}^{2}\right)\right]\right\} \end{array}$$

where Z is the normalization constant. Obviously, it can be seen that $b\left({𝔃}_{i,n}\right)$ is not a Gaussian function about ${𝔃}_{i,n}$, and it is difficult to transmit directly between nodes. To reduce communication overhead, Reference [19] approximates $b\left({𝔃}_{i,n}\right)$ by minimizing KL divergence:

(24) $$b\left({𝔃}_{i,n}\right)=\underset{q\left({𝔃}_{i,n}\right)\in 𝒢}{argmin}\mathrm{KLD}\left(q^{\left(m\right)}\left({𝔃}_{i,n}\right)|b^{\left(m\right)}\left({𝔃}_{i,n}\right)\right)$$

where 𝒢 represents a Gaussian function, and $\mathrm{KLD}\left(q\left({𝔃}_{i,n}\right)|b\left({𝔃}_{i,n}\right)\right)$ is the KL divergence between Gaussian function $q\left({𝔃}_{i,n}\right)$ and belief $b\left({𝔃}_{i,n}\right)$, the calculation formula is as follows:

(25) $$\begin{array}{l} \mathrm{KLD}\left(q\left({𝔃}_{i,n}\right)|b\left({𝔃}_{i,n}\right)\right)\\ =\frac{{∥x_{i,n}-x_{i,0}∥}^{2}+2\omega_{i,n}^{2}}{2\omega_{i,0}^{2}}-\ln\omega_{i,n}^{2}\\ +\sum_{j\in S_{i,n}}\ln\frac{-2\left({\tilde{\rho }}_{ij,n}-cTa_{ij,n}\right)\sqrt{\frac{\pi }{2}{-}_{i,n}^{2}}F\left(-\frac{1}{2},1,\frac{{∥x_{i,n}-x_{i,0}∥}^{2}}{2\sigma_{d}^{2}}\right)+∥x_{i,n}-x_{j,n}∥+2\sigma_{d}^{2}}{2\sigma_{d}^{2}}\\ +\sum_{k\in C_{i,n}}\ln\frac{-2\left({\tilde{\rho }}_{ik,n}-cTa_{ik,n}\right)\sqrt{\frac{\pi }{2}{-}_{i,n}^{2}}F\left(-\frac{1}{2},1,\frac{{∥x_{i,n}-x_{i,0}∥}^{2}}{2\sigma_{d}^{2}}\right)+∥x_{i,n}-x_{k,n}∥+2\sigma_{d}^{2}}{2\sigma_{d}^{2}} \end{array}$$

### 3.4. M-VMP-Based Joint Estimation Algorithm

**Figure 2:**
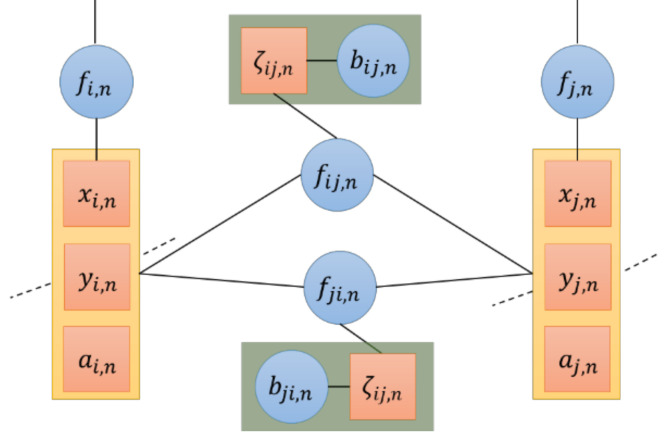
FG of a node pair (i,j) at time n. $x_{i,n},y_{i,n},a_{i,n}$ represent the localization and time synchronization parameter of the node to be estimated, $f_{i,n}$ represents the message that transmits between different times, $f_{ij,n}$ represents the distance information that the nodes transmit, $b_{ij,n}$ represents the non-sight distance parameter that affects the information between nodes and $\zeta_{ij,n}$ represents the non-sight distance error probability function that affects the non-sight distance parameter.

In Section 3.3, the clock drift of node or model loss with single Gaussian distribution are not considered, which affects localization accuracy [25]. In this paper, a joint estimation algorithm of time synchronization and localization based on multi-Gaussian distribution VMP is proposed, that the belief $b\left({𝔃}_{i,n}\right)$ of the variable ${𝔃}_{i,n}$ to be estimated is approximately a multi-Gaussian function $\hat{b}\left({𝔃}_{i,n}\right)\propto \sum \eta_{M,ij}𝒩\left({𝔃}_{k.i,n}|E_{{𝔃}_{i,n}},\;V_{{𝔃}_{i,n}}\right)$, and the belief of $\zeta_{ij,n}$ is $\hat{b}\left(\zeta_{ij,n}\right)\propto 𝒩\left(\zeta_{ij,n}|{\tilde{\rho }}_{ij,n}-cTa_{ij,n}-∥x_{i,n}-x_{j,n}∥,\sigma_{d}^{2}\right)$. The factor graph between nodes i and j at the time n is shown in Figure 2.

In order to approximate $b\left({𝔃}_{i,n}\right)$ to a Gaussian function, Formula (23) is rewritten as follows:

(26) $$b\left({𝔃}_{i,n}\right)\propto \left[f_{i,n}\left({𝔃}_{i,n}|{𝔃}_{i,n-1}\right)+\sum_{j\in S_{i,n}}f_{j}\left({𝔃}_{i,n}\right)+\sum_{k\in C_{i,n}}f_{k}\left({𝔃}_{i,n}\right)\right],$$

where:

(27) $$f_{i,n}\left({𝔃}_{i,n}|{𝔃}_{i,n-1}\right)≜\sum_{M}\frac{-\eta_{M,i}}{2}{\left({𝔃}_{i,n}-E_{{𝔃}_{i,n-1,M}}\right)}^{T}V_{i,n-1,M}^{-1}\left({𝔃}_{i,n}-E_{{𝔃}_{i,n-1,M}}\right)$$

(28) $$f_{j}\left({𝔃}_{i,n}\right)≜\sum_{M}-\eta_{M,ij}\frac{{\left({\tilde{\rho }}_{ij,n}-∥x_{i,n}-x_{j}∥-cTE_{a_{ij,n-1,M}}-b_{ij,n}\right)}^{2}}{2\sigma_{{𝔃}_{ij,n}}^{2}},$$

(29) $$f_{k}\left({𝔃}_{i,n}\right)≜\sum_{M}-\eta_{M,ik}\int \;b\left({𝔃}_{k,n}\right)\frac{{\left({\tilde{\rho }}_{ij,n}-∥x_{i,n}-x_{k,n}∥-cTE_{a_{ik,n-1,M}}-b_{ik,n}\right)}^{2}}{2\sigma_{{𝔃}_{ik,n}}^{2}}d{𝔃}_{k,n},$$

Because the existence of two non-linear terms, $∥x_{i,n}-x_{j}∥$ in $f_{j}\left({𝔃}_{i,n}\right)$ and $∥x_{i,n}-E_{x_{k,n-1,M}}∥$ in $f_{k}\left({𝔃}_{i,n}\right)$, results in non-Gaussian of $b\left({𝔃}_{i,n}\right)$, the two non-linear terms are expanded by a second-order Taylor expansion to obtain the mean and covariance of $b\left({𝔃}_{i,n}\right)$. For discussion purposes, define $g_{j}\left({𝔃}_{i,n}\right)≜∥x_{i,n}-x_{j}∥-cTE_{a_{ij,n-1,M}}$ and $g_{k}\left({𝔃}_{i,n},{𝔃}_{k,n}\right)≜∥x_{i,n}-x_{k,n}∥-cTE_{a_{ik,n-1,M}}$.

a. Second-order Taylor series expansion for nonlinear term $g_{j}\left({𝔃}_{i,n}\right):$$g_{j}\left({𝔃}_{i,n}\right)$ is expanded at ${𝔃}_{i,n}=E_{{𝔃}_{i,n-1}}$ by a second-order Taylor to get:
  (30) $$\begin{array}{ll} g_{j}\left({𝔃}_{i,n}\right) & =∥x_{i,n}-x_{j}∥-cTE_{a_{ij,n-1,M}}\\ \; & \approx g_{j}\left(E_{{𝔃}_{i,n-1}}\right)+\nabla^{T}g_{j}\left(E_{{𝔃}_{i,n-1}}\right)\left({𝔃}_{i,n}-E_{{𝔃}_{i,n-1}}\right)\\ \; & +\frac{1}{2}{\left({𝔃}_{i,n}-E_{{𝔃}_{i,n-1}}\right)}^{T}\nabla^{2}g_{j}\left(E_{{𝔃}_{i,n-1}}\right)\left({𝔃}_{i,n}-E_{{𝔃}_{i,n-1}}\right), \end{array}$$
  where $\nabla g_{j}\left(E_{{𝔃}_{i,n-1}}\right)$ and $\nabla^{2}g_{j}\left(E_{{𝔃}_{i,n-1}}\right)$ are the first and second steps of $g_{j}\left({𝔃}_{i,n}\right)$ at $E_{{𝔃}_{i,n-1}}$.
b. Second-order Taylor series expansion for nonlinear term $g_{k}\left({𝔃}_{i,n},{𝔃}_{k,n}\right)$: $g_{k}\left({𝔃}_{i,n},{𝔃}_{k,n}\right)$ is expanded at ${𝔃}_{i,n}=E_{{𝔃}_{i,n-1}}$ and ${𝔃}_{k,n}=E_{{𝔃}_{k,n-1}}$ by a second-order Taylor to get:
  (31) $$\begin{array}{cc} g_{k}\left({𝔃}_{i,n},{𝔃}_{k,n}\right) & =∥x_{i,n}-x_{k,n}∥-cTE_{a_{ik,n-1,M}}\\ \; & \approx g_{k}\left(E_{{𝔃}_{i,n-1}},E_{{𝔃}_{k,n-1}}\right)+\frac{\partial g_{k}\left(E_{{𝔃}_{i,n-1},n_{{𝔃}_{k,n-1}}}\right)}{\partial {𝔃}_{i,n}}\left({𝔃}_{i,n}-E_{{𝔃}_{i,n-1}}\right)\\ \; & +\frac{\partial^{T}g_{k}\left(E_{{𝔃}_{i,n-1}},E_{{𝔃}_{k,n-1}}\right)}{\partial {𝔃}_{k,n}}\left({𝔃}_{k,n}-E_{{𝔃}_{k,n-1}}\right)\\ \; & +\frac{1}{2}{\left(\begin{array}{c} {𝔃}_{i,n}-E_{{𝔃}_{i,n-1}}\\ {𝔃}_{k,n}-E_{{𝔃}_{k,n-1}} \end{array}\right)}^{T}\nabla^{2}g_{k}\left(E_{{𝔃}_{i,n-1}},E_{{𝔃}_{k,n-1}}\right)\left(\begin{array}{c} {𝔃}_{i,n}-E_{{𝔃}_{i,n-1}}\\ {𝔃}_{k,n}-E_{{𝔃}_{k,n-1}} \end{array}\right), \end{array}$$
  where $\frac{\partial g_{k}\left(E_{{𝔃}_{i,n-1}},E_{{𝔃}_{k,n-1}}\right)}{\partial {𝔃}_{i,n}}$ and $\frac{\partial g_{k}\left(E_{{𝔃}_{i,n-1}},E_{{𝔃}_{k,n-1}}\right)}{\partial {𝔃}_{k,n}}$ are the first-order partial derivatives of $g_{k}\left({𝔃}_{i,n},{𝔃}_{k,n}\right)$ with respect to ${𝔃}_{i,n}$ and ${𝔃}_{k,n}$ at ${𝔃}_{i,n}=E_{{𝔃}_{i,n-1}}$, and ${𝔃}_{k,n}=E_{{𝔃}_{k,n-1}}$, $\nabla^{2}g_{k}\left(E_{{𝔃}_{i,n-1}},E_{{𝔃}_{k,n-1}}\right)$ is the Hessian matrix of $g_{k}\left({𝔃}_{i,n},{𝔃}_{k,n}\right)$ at ${𝔃}_{i,n}=E_{{𝔃}_{i,n-1}}$ and ${𝔃}_{k,n}=E_{{𝔃}_{k,n-1}}$.

After calculation, we can get the mean $E_{{𝔃}_{i,n}}$ and covariance $V_{{𝔃}_{i,n}}$ of $b\left({𝔃}_{i,n}\right)$:

(32) $$\begin{array}{cc} V_{z_{i,n}}=\left[V_{z_{i,n-1}}^{-1}+\right. & \sum_{j\in S_{i,n}}\sum_{M_{1}}\frac{\eta_{M_{1},ij}}{\sigma_{d}^{2}}\left(I-\left({\tilde{\rho }}_{ij,n}-cTE_{a_{ij,n-1}}-\zeta_{ij,n}\right)\nabla^{2}g_{j}\left(E_{z_{i,n-1}}\right)\right)\\ \; & +\sum_{k\in C_{i,n}}\sum_{M_{2}}\frac{\eta_{M_{2},ik}}{\sigma_{d}^{2}}(I\\ \; & {\left.\left.-\left({\tilde{\rho }}_{ik,n}-cTE_{a_{ik,n-1}}-\zeta_{ik,n}\right)\nabla^{2}g_{k}\left(E_{z_{i,n-1}},E_{z_{k,n-1}}\right)\right)\right]}^{-1}, \end{array}$$

(33) $$\begin{array}{c} {\hat{E}}_{z_{i,n}}=V_{z_{i,n}}\left\{V_{z_{i,n-1}}^{-1}E_{z_{i,n-1}}\right.\\ \;\;\;\;\;\;\;+\sum_{j\in S_{i,n}}\sum_{M_{1}}\frac{\eta_{M_{1},ij}}{\sigma_{d}^{2}}\left(x_{j}\right.\\ \;\;\;\;\;\;\;\left.-\left({\tilde{\rho }}_{ij,n}-cTE_{a_{ij,n-1}}-\zeta_{ij,n}\right)\left(\nabla g_{j}\left(E_{z_{i,n-1}}\right)-\nabla^{2}g_{j}\left(E_{z_{i,n-1}}\right)E_{z_{i,n-1}}\right)\right)\\ \;\;\;\;\;\;\;+\sum_{k\in C_{i,n}}\sum_{M_{2}}\frac{\eta_{M_{2},ik}}{\sigma_{d}^{2}}\left(x_{k}\right.\\ \;\;\;\;\;\;\;-\left({\tilde{\rho }}_{ik,n}-cTE_{a_{ik,n-1}}-\zeta_{ik,n}\right)\left(\nabla g_{j}\left(E_{z_{i,n-1}}\right)\right.\\ \;\;\;\;\;\;\;\left.\left.-\nabla^{2}g_{k}\left(E_{z_{i,n-1}},E_{z_{k,n-1}}\right)E_{z_{i,n-1}}\right)\right)\}, \end{array}$$

where $\eta_{M,ij}=Z/\left(\rho_{ij}\cdot tr\left(V_{{𝔃}_{j,n-1}}\right)\right)$, and Z is the normalization constant. Compared with the approximation method based on KL divergence minimization, the proposed method based on multi-Gaussian approximation with second-order Taylor series expansion greatly reduces the complexity of approximate calculation. When $b\left({𝔃}_{i,n}\right)$ is approximated to a Gaussian function $\hat{b}\left({𝔃}_{i,n}\right)$, each node only needs to send its own position vector and covariance matrix to its neighbor nodes, and the communication overhead is much lower than that of the particle message-based method. In addition, since each node has three parameters to be estimated, the network is required to include at least three anchor nodes. The flow of the M-VMP localization algorithm (Algorithm 1) is as follows:
**Algorithm 1**: M-VMP joint estimation algorithmInitialization:  Initialize node location distribution   $f_{i,0}p\left({𝔃}_{i,0}\right)$Location estimation:for n = 1 to N (time index) do  Nodes i∈C    1) Compute covariance $V_{{𝔃}_{i,n}}$ of belief $\hat{b}\left({𝔃}_{i,n}\right)$ according to (32)    2) Compute mean ${\hat{E}}_{z_{i,n}}$ of belief $\hat{b}\left({𝔃}_{i,n}\right)$ according to (33)    3) Broadcast means and variances of belief $\hat{b}\left({𝔃}_{i,n}\right)$, and receive the mean     and covariance matrix of location variables of neighbor nodes    4) Estimate location information of node i according to MMSE principle    5) Update location information and form messages $\mu_{{𝔃}_{i,n}\to f_{i,n+1}}$  end parallelend for


## 4. Simulation Analysis and Test Results

In this section, Cramer-Rao Lower Bound (CRLB) of ${𝔃}_{i,n}$ is derived first, then the performance of the proposed joint algorithms is analyzed through simulation.

### 4.1. CRLB Lower Bound of ${𝔃}_{i,n}$

For node i, the Fisher Information Matrix $F\left({𝔃}_{i,n}\right)$ can be derived as:

(34) $$F\left({𝔃}_{i,n}\right)=A_{i}diag\left(\ldots ,\sigma_{d}^{2},\ldots \right)A_{i}^{T},$$

with

(35) $$A_{i,n}=\left[\ldots ,u_{ij,n},\ldots \right],j\in N_{i,n}$$

(36) $$u_{ij,n}=\frac{1}{\rho_{ij,n}}\left[\begin{array}{c} x_{i,n}-x_{j,n}\\ y_{i,n}-y_{j,n}\\ cTa_{ij,n} \end{array}\right],$$

and CRLB of ${𝔃}_{i,n}$ is $\mathrm{CRLB}\left({𝔃}_{i,n}\right)=F^{-1}\left({𝔃}_{i,n}\right)$.

### 4.2. Simulation Scenario and Result Analysis

**Figure 3:**
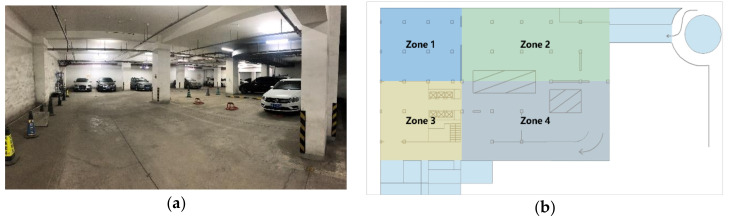
(**a**) The real scene of the underground parking lot. (**b**) Simulation scenario. Zone 1 is the simulation area.

To analyze the performance of the proposed method, we have built a simulation scenario according to the real scene of the zone 1, underground parking lot, Beijing University of Posts and Telecommunications. The real scene is shown in Figure 3a and the top view is shown in Figure 3b.

According to the actual size of zone 1, the simulation scene is a rectangular area of 20 × 24 m. By default, the number of anchor nodes is 4, the number of nodes to be located is 20 and the initial position distribution of nodes in the site conforms to the uniform distribution. In order to limit the communication area of nodes to less than 1/2 of the whole simulation area, the maximum communication distance is set to 10 m. The performance of the proposed method is obtained in the line of sight (LOS) environment, except the last simulation, which shows localization accuracy with different NLOS probability. All simulation results are the average of 1000 independent runs.

The initial position measurement error of the anchor node conforms to the Gaussian distribution with the standard deviation of 0.1 m. The initial position measurement error distribution of the node to be located is composed of Gaussian distributions with the standard deviation of 10 m. According to the performance of the crystal oscillator used in the hardware (TG5032CFN), the clock drift of the node to be located conforms to uniform distribution from [−1 ppm, 1 ppm], that is, the maximum distance measurement error caused by the clock drift between adjacent time (1 s) is 300 m. In the process of simulation, anchor nodes remain stationary, and the velocity of the node to be located is consistent with the uniform distribution of the maximum value of 3 m/s, and the direction is random every time step. The position movement measurement of the node conforms to the Gaussian distribution with the standard deviation of 1 m. The default process is 15 s, 10 iterations per second. Root mean square error (RMSE) and cumulative distribution functions (CDF) are used to measure the performance of algorithms.

**Figure 4:**
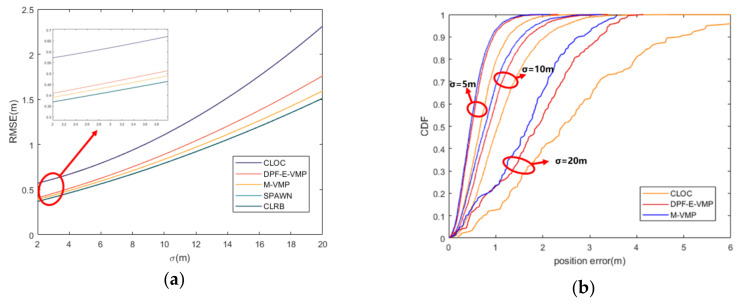
(**a**) Root mean square error (RMSE) of position error with different initial location error. (**b**) Cumulative distribution function (CDF) of position error with different initial location error.

In Figure 4a, the relationship between localization RMSE and initial location error is shown. The CLOC method [16] treats the uncertainties of nodes’ positions and clock offsets as measurement noise and thus suffers performance degradation. The SPAWN [26] method is implemented by using 4000 particles to represent the messages on FG. The localization accuracy of the VMP method [17] and the proposed M-VMP method are better than the CLOC method, but slightly worse than the SPAWN method.

The position error of VMP and the proposed M-VMP method are compared in Figure 4b. The cumulative distribution function of the VMP method and the proposed method under different initial position errors is given in Figure 4b. It is found that the probability of position error less than 1.6 m of the two methods under different initial position errors is basically the same, while the proposed method has a better effect of restraining position error. The result is consistent with the conclusion in Figure 4a.

**Figure 5:**
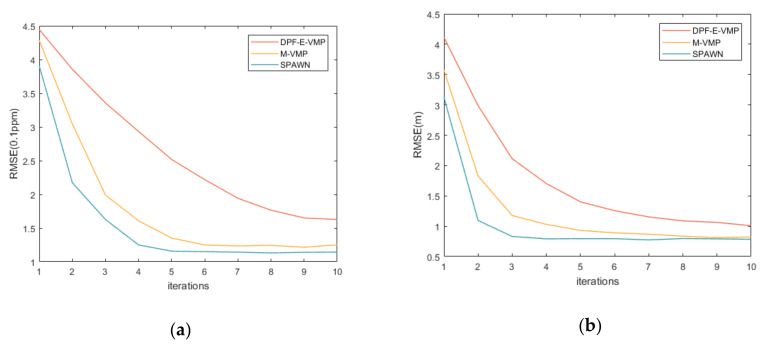
(**a**) RMSE of the clock drift slope versus iterations and (**b**) RMSE of position error versus iterations.

Figure 5 shows the relationship among the clock drift slope, RMSE of localization accuracy and the number of iterations, respectively. It is seen that three methods converge in the finite iterations, which proves that the algorithms are feasible. Compared with the VMP method [17], the proposed method improves the convergence speed of localization and time synchronization by adding time synchronization parameters into the localization process.

**Figure 6:**
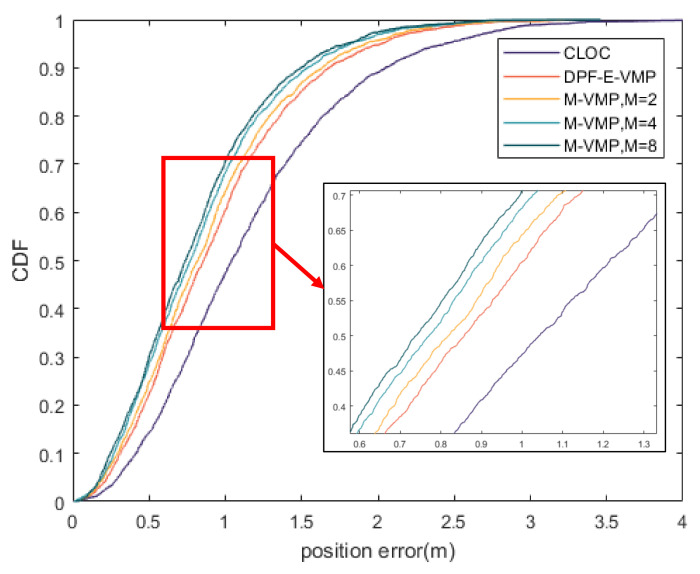
CDF of position error with different values of the number of Gaussian distribution M in line of sight (LOS) environment.

In Figure 6, the CDF of the proposed method under different values of the number of Gaussian distribution M are compared. It is seen that the localization accuracy (3σ) of the proposed method is better than that of the VMP method when M is greater than 2. With the increase of M, the localization accuracy is also improved.

**Figure 7:**
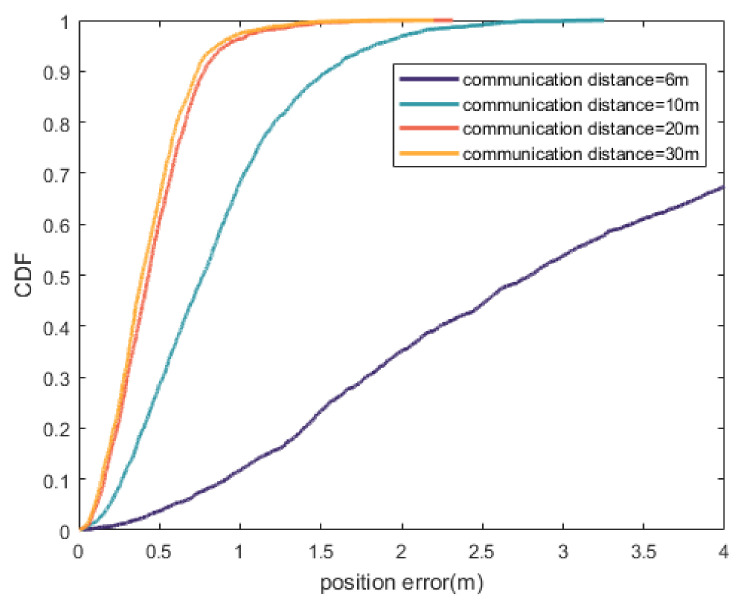
CDF of position error with different communication distance.

Figure 7 reflects the influence of communication distance on the localization accuracy of the proposed method under the same node density. Obviously, localization accuracy increases with the increase of communication distance, which increases the connectivity of nodes and the redundancy of information. At the same time, it can be seen that the improvement of localization accuracy is not obvious when the communication distance is 20 and 30 m. The difference of node connectivity between those two conditions is little, so the localization accuracy cannot be greatly improved. Because the communication distance is proportional to the transmission power of the node, it is necessary to determine the appropriate communication distance in practical application by considering the localization accuracy, node power consumption and other indicators.

**Figure 8:**
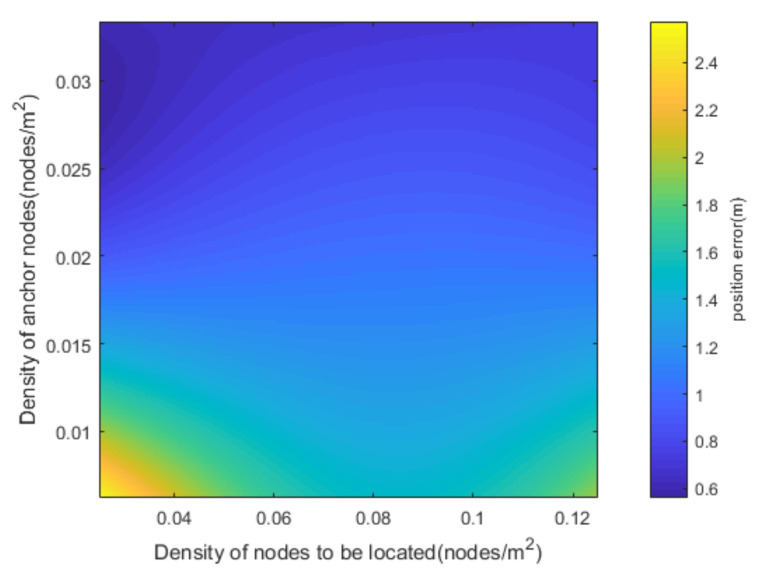
Relationship between position error and nodes’ density.

In the simulation of Figure 8, in order to amplify the influence of node density on positioning accuracy, we reset some of the simulation parameters, and set the communication distance to 15 m and the variance of the distance observation and the initial position error of the node to be located to 20 m^2^. It can be seen from Figure 8 that as the density of nodes increases, the overall positioning accuracy is decreasing. However, when the density of nodes to be located is too large, the positioning error will increase. At the same time, when there are more nodes to be located, the speed at which the positioning error decreases with more anchor nodes also decreases. This is due to the increase in the density of nodes to be located, and the overall weight of the information contained in neighboring nodes to be located in the proposed method becomes larger. When the number of anchor nodes is insufficient, the increase of neighboring nodes to be located helps to improve the positioning accuracy, but the effect is limited.

**Figure 9:**
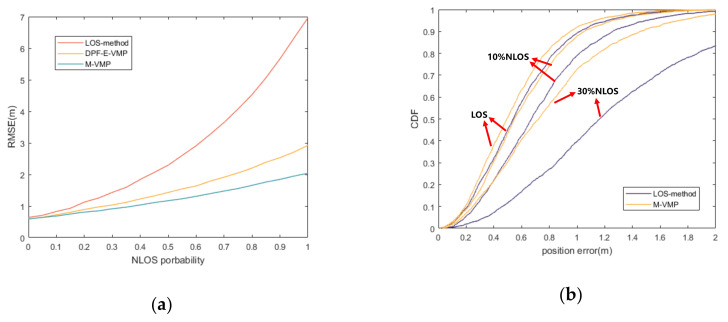
(**a**) RMSE of position accuracy with different NLOS probability. (**b**) CDF of position error with different NLOS probability.

In Figure 9, the relationship between the RMSE of localization accuracy and NLOS occurrence probability of the VMP method and the proposed method is compared. Furthermore, it compares with the method that only deals with LOS. It is seen that both the VMP and the proposed method effectively suppress NLOS error. Because the two-way NLOS parameters between nodes are added in the proposed method, the NLOS error is better suppressed.

### 4.3. Computational Complexity and Communication Overhead Analysis

Because the proposed method in this paper and the comparison methods are distributed algorithms, the computation and communication are done by the nodes independently, so only one node’s time complexity and communication overhead need to be considered in the analysis.

The time complexity is evaluated by the number of operations in local computing, and the communication cost is evaluated by the number of information parameters broadcast by nodes. The time complexity of the CLOC method is related to its neighbor nodes number $N_{\mathrm{inb}}$, and its communication cost is $3\cdot O\left(1\right)$. The SPAWN method is based on particles. When the number of particles in each message is $N_{p}$, its time complexity is $O\left(N_{p}+N_{p}^{2}\cdot N_{\mathrm{inb}}\right)$, and its communication cost is $O\left(N_{p}\right)+2\cdot O\left(1\right)$. Compared with the VMP method, the number of Gaussian messages in the proposed method is M times, and every message from neighbor nodes needs to be processed once. The time complexity of dimension reduction depends on $N_{\mathrm{inb}}$, so its time complexity is $O\left(M\cdot N_{\mathrm{inb}}\right)+O\left(N_{\mathrm{inb}}\right)$. In the proposed method, nodes transmit a localization vector including $x_{i,n},y_{i,n},a_{i,n}$ and a covariance matrix, so the communication cost is $3O\left(M\right)+O\left(1\right)$. The computational complexity, run-time and communication Overhead of the three algorithms is shown in Table 2.

### 4.4. Future Research Directions

In the next stage of research, we will mainly focus on the following aspects: First, analyze the performance of the positioning and simultaneous joint estimation problem and its influencing factors in principle. Secondly, expand the positioning scene from 2 dimensions to 3 dimensions, and reduce the problem of positioning accuracy degradation in dense scenes with nodes to be located through methods such as signal quality screening. Finally, the proposed algorithm will be implemented based on the hardware platform, and the measured results will be compared with the simulation results to further improve the performance of the proposed method.

## 5. Conclusions

This paper presented a M-VMP-based TOA localization and time synchronization joint estimation algorithm for mixed LOS and NLOS environments. Firstly, according to the VMP method, a message propagation model based on a factor graph was constructed for localization and time synchronization. Owing to the existence of nonlinear terms, it is difficult to represent the message in a closed form, so Taylor expansion was used for the linearization of nonlinear terms. Moreover, to reduce the communication cost, all messages were expressed in the form of multi-Gaussian distribution, and only the mean value, covariance and weight of each Gaussian distribution need to be transferred in the message transmission. The simulation results showed that the accuracy and convergence speed of the proposed method were close to that of SPAWN method, and the time complexity and communication cost were greatly reduced: the run-time was only 1.5% that of SPAWN.

## Figures and Tables

**Table 1:** List of symbols.

Symbol	Meaning	Symbol	Meaning
$S_{i,n}$	Set of neighbor anchor nodes of node i at time n	$C_{i,n}$	Set of neighbor nodes to be located of node i at time n
$x_{i,n}$	Position vector of node i at time n	${\tilde{t}}_{i,n}$	Measurement value of local clock of note i at time n
$T_{n}$	Real time value at time n	$a_{i,n}$	Slope of local clock of node i at time n
$a_{ij,n}$	Relative slope of local clock offset between nodes i,j	$N_{i,n}$	Set of neighbor nodes of node i at time n
${\tilde{\rho }}_{ij,n}$	Range measurement between node i,j at time n	$\omega_{ij,n}$	Measurement noise of ${\tilde{\rho }}_{ij,n}$
$\sigma_{d}^{2}$	Variance of $\omega_{ij,n}$	$\zeta_{ij,n}$	NLOS error of ${\tilde{\rho }}_{ij,n}$
$\Psi_{n}$	Set of all communicable node pairs $\left(i,j\right)$ at time n	$\Theta_{n}$	Set of all node pairs $\left(i,j\right)$ with NLOS error at time n
$x_{n}$	Position vector of all nodes at time n	$a_{n}$	Clock offset of all nodes at time n
$v_{i,n}$	Average velocity of node i from time n−1 to time n	$\omega_{i,n}$	Measurement noise of $v_{i,n}$
$\sigma_{i,n}^{2}$	Variance of $\omega_{i,n}$	${𝔃}_{i,n}$	Vector to be estimated of note i at time n
${\hat{𝔃}}_{i,n}$	Estimation result of node i at time n	${\tilde{\rho }}_{n}$	Range measurement in all node pairs $\left(i,j\right)\in V_{n}$ at time n
$\zeta_{n}$	NLOS error in all node pairs $\left(i,j\right)\in \Theta_{n}$ at time n	${𝓧}_{1:n},{𝓐}_{1:n},{𝓟}_{1:n},{𝓑}_{1:n}$	Vector sets of $x_{n},a_{n},{\tilde{\rho }}_{n},\zeta_{n}$ from time 1 to time n
$\mu_{f_{ij,n}\to {𝔃}_{i,n}}\left({𝔃}_{i,n}\right)$	Message send from $f_{ij,n}$ to ${𝔃}_{i,n}$	$b\left({𝔃}_{i,n}\right)$	Belief of variable ${𝔃}_{i,n}$
$F\left(\cdot \right)$	Confluent Hypergeometric Function of the First Type	$E_{{𝔃}_{i,n}}$	Mean of belief $b\left({𝔃}_{i,n}\right)$
$V_{{𝔃}_{i,n}}$	Covariance of belief $b\left({𝔃}_{i,n}\right)$	$\eta_{M,ij}$	Weight of the M-th Gaussian distribution
$F\left({𝔃}_{i,n}\right)$	Fisher Information Matrix (FIM) of ${𝔃}_{i,n}$	$\mathrm{CRLB}\left({𝔃}_{i,n}\right)$	Cramer-Rao Lower Bound of ${𝔃}_{i,n}$

**Table 2:** Comparisons of different methods for each node at each iteration.

Method	Computational Complexity	Run-Time	Communication Overhead
M-VMP (proposed method)	$O\left(M\cdot N_{inb}\right)+O\left(N_{inb}\right)$	84.449 ms	$3\cdot O\left(M\right)+O\left(1\right)$
CLOC	$O\left(N_{inb}^{2}\right)$	288.500 ms	$3\cdot O\left(1\right)$
SPAWN	$O\left(N_{p}+N_{p}^{2}\cdot N_{inb}\right)$	5469.089 ms	$O\left(N_{p}\right)+2\cdot O\left(1\right)$
